# Keishibukuryogan, a Traditional Japanese Medicine, Inhibits Platelet Aggregation in Guinea Pig Whole Blood

**DOI:** 10.1155/2015/295706

**Published:** 2015-08-24

**Authors:** Kiyoshi Terawaki, Masamichi Noguchi, Mitsutoshi Yuzurihara, Yuji Omiya, Yasushi Ikarashi, Yoshio Kase

**Affiliations:** Tsumura Research Laboratories, Kampo Scientific Strategies Division, Tsumura & Co., 3586 Yoshiwara, Ami-machi, Inashiki-gun, Ibaraki 300-1192, Japan

## Abstract

Effects of keishibukuryogan (KBG) on platelet aggregation were investigated. To ensure the specificity of KBG, tokishakuyakusan (TSS) and kamisyoyosan (KSS), which are known to have platelet aggregation-inhibiting effects, and rikkunshito (RKT) and shakuyakukanzoto (SKT), which are considered to be devoid of such effects, were used for comparison. The platelet aggregation of each test drug was measured by the screen filtration pressure method using whole blood of guinea pigs and expressed as a collagen-induced pressure rate (%) or a collagen concentration required for 50% increase in the pressure rate (PATI value). KBG suppressed the collagen-induced whole blood pressure rate increase and increased the PATI value, like TSS and KSS. Neither RKT nor SKT showed these effects. The Moutan cortex and Cinnamomi cortex, the constituent crude drugs of KBG, showed KBG-like pressure rate suppression and PATI-increasing effects. Furthermore, paeonol, a representative component of Moutan cortex, and aspirin which is known to have platelet aggregation-inhibiting activity (COX-1 inhibitor) also showed similar effects. These results suggest that the platelet aggregation-inhibiting activity of the constituent crude drugs Moutan cortex and Cinnamomi cortex is involved in the improving effects of KBG on impaired microcirculation and that paeonol plays a role in these effects.

## 1. Introduction

The microcirculatory system is a type of vascular bed including arterioles and capillaries and accounts for more than 90% of the systemic vascular system. Its impairment is related not only to cardiovascular disorders but also to vascular complications of diabetes, hepatic and renal dysfunction, and obstetric and gynecologic conditions such as the oversensitivity to cold. In microcirculatory disturbances, endothelial cells of the vascular wall are damaged, and leukocytes or platelets agglutinate on endothelial cells to form a clot. In addition, the disturbances are also caused by various factors such as increased blood viscosity, active oxygen species, decreased erythrocyte deformability, coagulation/fibrinolysis system, and adhesion factors. In Kampo medicine (traditional Japanese medicine), these complex microcirculatory disturbances are referred to as oketsu (impaired microcirculation) [1, 2]. Keishibukuryogan (KBG), tokishakuyakusan (TSS), and kamisyoyosan (KSS) have been used as oketsu-improving drugs (antioketsu drugs), with KBG particularly showing potent improving effects [3, 4]. It has been suggested that platelet aggregation-inhibiting activity is involved in the improving effects of TSS and KSS on microcirculatory disturbances [5]. However, the effects of KBG on platelet aggregation have been little investigated [6], although increased blood viscosity and reduced erythrocyte deformability are reported to be involved in the antioketsu effects [3, 7].

While platelet-rich plasma (PRP) has been used in platelet aggregation tests [8], PRP has been shown to have effects on the platelet activation and the sensitivity to coagulation stimuli because it is adjusted by centrifugation used in its preparation from whole blood. For this reason, attempts are being made to directly test platelet aggregation using whole blood [9–12]. Moreover, platelet aggregation measured with guinea pig or mouse whole blood has been shown to be similar to that measured with human whole blood [13].

In this study, we used the screen filtration pressure (SFP) method [9–11, 13, 14] with guinea pig whole blood to investigate the mechanistic involvement of platelet aggregation-inhibiting action in the microcirculation disorder-improving effects of KBG and to identify active crude drugs and components contributing to these effects. In addition, TSS and KSS which are known to have platelet aggregation-inhibiting effects [5] and rikkunshito (RKT; a upper gastrointestinal disorder-improving drug) [15–18] and shakuyakukanzoto (SKT; a muscle cramp-improving drug) [19–21], which do not have such effects, were used for comparison to ensure the specificity of KBG.

## 2. Materials and Methods

### 2.1. Animals

Seven-week-old male Hartley guinea pigs weighing 400–500 g were obtained from Japan SLC Inc. (Hamamatsu, Japan). The animals were allowed free access to water and a standard laboratory food, CG7 (Clea, Tokyo, Japan). They were kept in a facility at a temperature of 24 ± 1°C, relative humidity of 55 ± 5%, and with lights on from 07:00 to 19:00 daily. All experimental procedures were performed according to the “Guidelines for the Care and Use of Laboratory Animals” approved by the Laboratory Animal Committee of Tsumura & Co.

### 2.2. Drugs and Reagents

The powdered extracts of KBG (Lot number 241059300), TSS (Lot number 2020023010), KSS (Lot number 2040024010), RKT (Lot number 2020043010), SKT (Lot number 280068010), and the five constituents crude drugs of KBG, Moutan cortex (Lot number 2061006010), Hoelen (Lot number 2061007010), Cinnamomi cortex (Lot number 2061003010), Persicae semen (Lot number 2061012010), and Paeoniae radix (Lot number 2061001010) were supplied by Tsumura & Co. (Tokyo, Japan).

KBG is a mixture of five constituent crude drugs: 3.0 g Cinnamomi cortex (bark of* Cinnamomum cassia* Blume, Lauraceae), 3.0 g Paeoniae radix (root of* Paeonia lactiflora* Pallas, Paeoniaceae), 3.0 g Persicae semen (seed of* Prunus persica* Batsch, Rosaceae), 3.0 g Hoelen (sclerotium of Poria cocos Wolf, Polyporaceae), and 3.0 g Moutan cortex (bark of* Paeonia suffruticosa* Andrews, Paeoniaceae).

TSS is a mixture of six constituent crude drugs: 4.0 g Paeoniae radix, 4.0 g Atractylodis lanceae rhizoma (rhizome of* Atractylodes lancea* De Candolle, Compositae), 4.0 g Alismatis rhizoma (rhizome of* Alisma orientale* Juzepczuk, Alismataceae), 4.0 g Hoelen, 3.0 g Cnidii rhizoma (rhizome of* Cnidium officinale* Makino, Umbelliferae) and 3.0 g Angelicae radix (root of* Angelica acutiloba* Kitagawa, Umbelliferae).

KSS is a mixture of ten constituent crude drugs: 3.0 g Bupleuri radix (root of* Bupleurum falcatum* Linné, Umbelliferae), 3.0 g Paeoniae radix, 3.0 g Atractylodis lanceae rhizoma, 3.0 g Angelicae radix, 3.0 g Hoelen, 2.0 g Gardeniae fructus (fruit of* Gardenia jasminoides* Ellis, Rubiaceae), 2.0 g Moutan cortex, 1.5 g Glycyrrhizae radix (root of* Glycyrrhiza uralensis* Fischer, Leguminosae), 1.0 g Zingiberis rhizoma (root of* Zingiber officinale* Roscoe, Zingiberaceae), and 1.0 g Menthae Herba (above ground parts of* Mentha arvensis* Linné var.* piperascens* Malinvaud, Labiatae).

RKT is a mixture of eight constituent crude drugs: 4.0 g Atractylodis lanceae rhizoma, 4.0 g Ginseng radix (root of* Panax ginseng* C. A. Meyer, Araliaceae), 4.0 g Pinelliae Tuber (tuber of* Pinellia ternata* Breitenbach, Araceae), 4.0 g Hoelen, 2.0 g Zizyphi fructus (fruit of* Zizyphus jujuba* Miller var.* inermis* Rehder), 2.0 g Aurantii nobilis pericarpium (peel of* Citrus unshiu* Markovich, Rutaceae), 1.0 g Glycyrrhizae radix, and 0.5 g Zingiberis rhizome.

SKT is a mixture of two constituent crude drugs: 6.0 g Glycyrrhizae radix and 6.0 g Paeoniae radix.

Each mixture of constituent crude drugs of KBG, TSS, KSS, RKT, and SKT was extracted with purified water at 95°C for 1 h, and the extraction solution was separated from the insoluble waste and concentrated by removing water under reduced pressure. Spray-drying was used to produce a dried extract powder.

Paeonol and aspirin (acetylsalicylic acid) were purchased from Wako Pure Chemical Industries, Ltd. (Osaka, Japan). Collagen solution was purchased from MC Medical Inc. (Tokyo, Japan). The other reagents used for analysis were purchased from commercial sources.

KBG, TSS, KSS, RKT, SKT, and five constituent crude drugs of KBG were dissolved separately in methanol and sonicated at 25°C for 30 min. The solution was centrifuged at 900 ×g for 3 min, and the supernatant was filtered through a 0.45 *μ*m filter. Paeonol and aspirin were dissolved in methanol at the final concentration of 10–50 *μ*g/mL and 100 *μ*M, respectively.

### 2.3. Measurement of Platelet Aggregation by Screen Filtration Pressure

Platelet aggregation was measured with a whole blood aggregometer, WBA (MC Medical Inc., Tokyo, Japan), using a screen filtration pressure method [13, 22]. For preparation of blood samples, whole blood was collected from the inferior vena cava of guinea pigs under ether anesthesia. Immediately, 3.18% trisodium citrate was added to collected blood in the ratio of 1 : 9 (v/v). Then, blood samples were stored at room temperature for 1 h before assay. Five-*μ*L of test substance solution dissolved in methanol or vehicle as a control was added to 195 *μ*L of whole blood in a reaction tube (methanol concentration: 2.5%). The blood containing test substance was stirred constantly with a stirring bar, in an incubator at 37°C for 5 min. After incubation, 22.2 *μ*L of collagen solution (final concentration: 1, 2, 4, or 8 *μ*g/mL) was added to the reaction tube. After incubation for 5 min, filter-unit syringes with screen microsieves made of nickel and containing three hundred 30 *μ*m square openings per 1 mm diameter area (code number SSR4421, MC Medical Inc.) were connected to a pressure sensor, and the blood samples were automatically aspirated into the syringes. A negative pressure of −130 mmHg was established as 100%, and −6 mmHg, rather than 0 mmHg, was used as the 0% pressure rate to compensate for the viscosity of whole blood. The platelet aggregation pressure of each reaction tube was determined as the pressure rate (%). The collagen concentration (*μ*g/mL) at 50% pressure was expressed as the platelet aggregatory threshold index (PATI) values. Because the upper limit of determination of PATI was 8 *μ*g/mL, PATI values above 8 *μ*g/mL were expressed as 8 *μ*g/mL.

### 2.4. Statistical Analyses

Each value was expressed as the mean ± SEM. Statistical analysis of the pressure rates was evaluated by a one-way analysis of variance (ANOVA) coupled with Dunnett's multiple comparison test. The response curve of the pressure rates and PATI values were evaluated by two-factor factorial ANOVA followed by a post hoc Bonferroni multiple comparison test and the unpaired Student's *t*-test, respectively. Significance was accepted at *p* < 0.05.

## 3. Results

### 3.1. Effects of KBG on Collagen-Induced Platelet Aggregation

**Figure 1:**
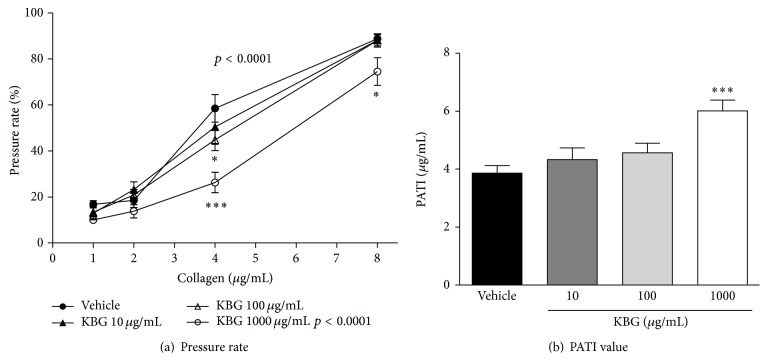
Effects of KBG on collagen-induced platelet aggregation of guinea pig whole blood. The results on pressure rate and PATI values were shown in (a) and (b). Data are expressed as the mean ± SEM (*n* = 12 animals). Significant differences in (a) data was evaluated using two-way ANOVA followed by a post hoc Bonferroni multiple comparison test. Significant difference was observed in 1000 *μ*g/mL-KGB group (*p* < 0.0001) compared to control group. Post hoc analysis was performed at each concentration of collagen: ^*∗*^ *p* < 0.05 and ^*∗∗∗*^ *p* < 0.001 versus vehicle. The data in (b) was evaluated using the unpaired Student's *t*-test: ^*∗∗∗*^ *p* < 0.001 versus vehicle.

Figure 1 shows the effects of KBG on collagen-induced platelet aggregation in whole blood.

Collagen (1–8 *μ*g/mL) increased the whole blood filtration pressure rate in a concentration-dependent manner, in vehicle control group. Compared to control group, factorial analysis indicated that there was a significant difference in 1000 *μ*g/mL KGB group (*p* < 0.0001). Post hoc analysis indicated that the collagen-induced increase in whole blood pressure rate was inhibited by the concomitant use of KBG in a concentration-dependent manner (10–1000 *μ*g/mL); that is, significant inhibitory effects were observed in the 100 and 1000 *μ*g/mL KBG-treated groups on the pressure rate increase induced by 4 *μ*g/mL of collagen and in the 1000 *μ*g/mL KBG-treated group on the pressure rate increase induced by 8 *μ*g/mL of collagen (Figure 1(a)). PATI values calculated from the results in Figure 1(a) are shown in Figure 1(b). A PATI value represents a concentration of collagen required for a 50% increase of the whole blood pressure rate elevation induced by platelet aggregation. In the control (vehicle) group, 3.86 *μ*g/mL of collagen was required to increase the pressure rate by 50% (PATI value). The PATI value increased upon the addition of KBG in a concentration-dependent manner. Significant increase was observed at 1000 *μ*g/mL. These results suggest that KBG suppresses the collagen-induced platelet aggregation measured as the whole blood pressure rate increases.

### 3.2. Effects of TSS, KSS, RKT, and SKT on Collagen-Induced Platelet Aggregation

**Figure 2:**
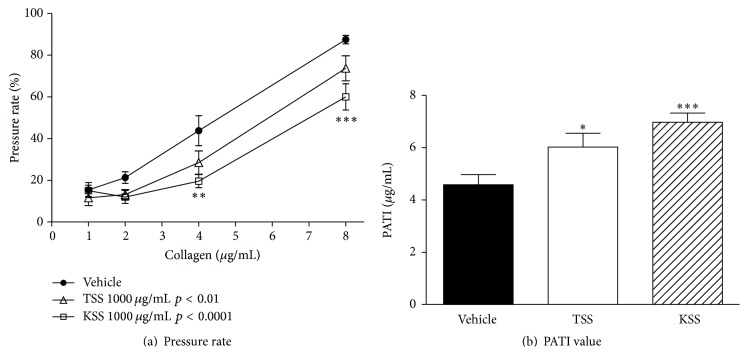
Effects of TSS and KSS on collagen-induced platelet aggregation of guinea pig whole blood. The results on pressure rate and PATI values were shown in (a) and (b). Data are expressed as the mean ± SEM (*n* = 9–11 animals). Significant differences in (a) data were evaluated using two-way ANOVA followed by a post hoc Bonferroni multiple comparison test. Significant difference was observed in TSS group (*p* < 0.01) and KSS group (*p* < 0.0001) compared to control group. Post hoc analysis was performed at each concentration of collagen: ^*∗∗*^ *p* < 0.01 and ^*∗∗∗*^ *p* < 0.001 versus vehicle. The data in (b) was evaluated using the unpaired Student's *t*-test: ^*∗*^ *p* < 0.05 and ^*∗∗∗*^ *p* < 0.001 versus vehicle.

Figure 2 shows the effects of TSS and KSS used as positive controls on collagen-induced platelet aggregation in whole blood. Factorial analysis indicated that there was a significant difference in TSS (*p* < 0.01) and KSS (*p* < 0.0001) groups compared to control group. In post hoc analysis, TSS (1000 *μ*g/mL) showed near-significant inhibitory effects on the whole blood pressure rate increases induced by 4 and 8 *μ*g/mL of collagen, and KSS (1000 *μ*g/mL) showed significant effects under the same conditions (Figure 2(a)). PATI values of the TSS and KSS groups were significantly increased compared with the value of the vehicle control group. These results suggest that both formulations have platelet aggregation-inhibiting effects (Figure 2(b)).

**Figure 3:**
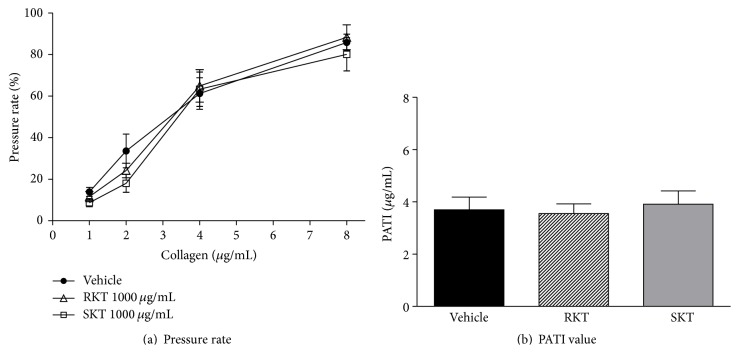
Effects of RKT and SKT on collagen-induced platelet aggregation of guinea pig whole blood. The results on pressure rate and PATI values were shown in (a) and (b). Data are expressed as the mean ± SEM (*n* = 11 animals). Significant differences in (a) data were evaluated using two-way ANOVA followed by a post hoc Bonferroni multiple comparison test. The data in (b) was evaluated using the unpaired Student's *t*-test. No significant differences were observed among groups in (a) and (b).

Figure 3 shows the effects of RKT and SKT used as negative controls in platelet aggregation assays. Neither RKT (1000 *μ*g/mL) nor SKT (1000 *μ*g/mL) significantly affected whole blood pressure rate increases induced by 1–8 *μ*g/mL of collagen (Figure 2(a)). In addition, they did not significantly alter the PATI values calculated from these results compared to the vehicle control group (Figure 3(b)).

### 3.3. Effects of Constituent Crude Drugs of KBG on Collagen-Induced Platelet Aggregation

**Figure 4:**
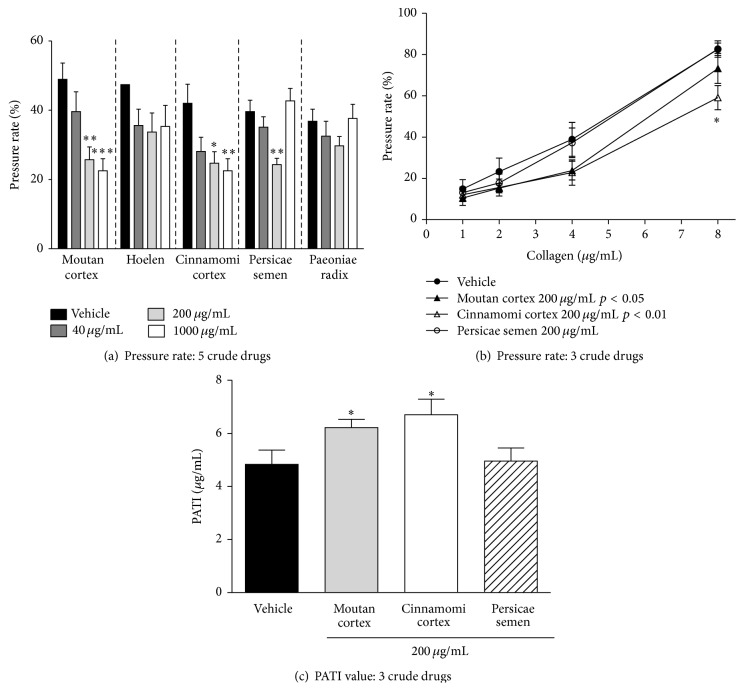
Effects of constituent crude drugs of KBG on collagen-induced platelet aggregation of guinea pig whole blood. (a) Effects of 5 crude drugs (40–1000 *μ*g/mL) on 4 *μ*g/mL collagen-induced increase in pressure rates. (b) Effects of 3 crude drugs (200 *μ*g/mL) on collagen-induced increase (1–8 *μ*g/mL) in pressure rates. (c) PATI values for 3 crude drugs. Data are expressed as the mean ± SEM (*n* = 9-10 animals). Significant differences in each group of (a) data were evaluated using one-way ANOVA followed by a post hoc Dunnett's multiple comparison test: ^*∗*^ *p* < 0.05, ^*∗*^ *p* < 0.01, and ^*∗∗∗*^ *p* < 0.001 versus vehicle. The data in (b) was evaluated using two-way ANOVA followed by a post hoc Bonferroni multiple comparison test. Significant difference was observed in Moutan cortex group (*p* < 0.05) and Cinnamomi cortex group (*p* < 0.01) compared to control group. Post hoc analysis was performed at each concentration of collagen: ^*∗*^ *p* < 0.05. The data in Figure 4(c) was evaluated using the unpaired Student's *t*-test: ^*∗*^ *p* < 0.05 versus vehicle.

To identify the active crude drugs involved in the inhibitory effect of KBG on collagen-induced platelet aggregation, 5 crude drugs (40–1000 *μ*g/mL) constituting the KBG were screened using the suppressive effect on the whole blood pressure rate increase induced by 4 *μ*g/mL of collagen as an indicator (Figure 4(a)). Moutan cortex and Cinnamomi cortex inhibited the collagen-induced whole blood pressure rate increase in concentration-dependent manners. Persicae semen suppressed the pressure rate in a concentration-dependent manner up to 200 *μ*g/mL, but no suppression was observed at a higher concentration (1000 *μ*g/mL). Paeoniae radix and Hoelen showed no significant effects on the collagen-induced pressure rate increase.

Next, we examined the effects of Moutan cortex, Cinnamomi cortex, and Persicae semen at a fixed concentration of 200 *μ*g/mL, on collagen-induced platelet aggregation (1–8 *μ*g/mL) in whole blood for determination of PATI values (Figures 4(b) and 4(c)). In Figure 4(b), factorial analysis indicated that the collagen-induced increase in whole blood pressure rate was significantly inhibited in Moutan cortex group (*p* < 0.05) and Cinnamomi cortex (*p* < 0.01) group compared with control group. However, no significant difference was observed between Persicae semen and control groups.

PATI values of Moutan cortex and Cinnamomi cortex were significantly increased (*p* < 0.05) compared with the value of the vehicle control group. However, no significant differences in the increased levels were observed between two groups (Figure 4(c)). No significant difference was observed between Persicae semen and control groups.

Taken together, these results (Figures 4(a), 4(b), and 4(c)) suggest that Moutan cortex and Cinnamomi cortex are, at least, responsible for the effect of KBG.

### 3.4. Inhibitory Effects of Paeonol and Aspirin on Platelet Aggregation

**Figure 5:**
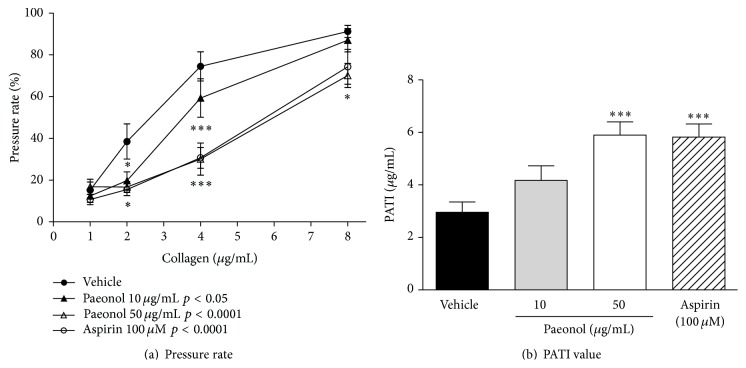
Effects of paeonol or aspirin on collagen-induced platelet aggregation of guinea pig whole blood. The results of pressure rate and PATI values were shown in (a) and (b). Data are expressed as the mean ± SEM (*n* = 10 animals). Significant differences in (a) data was evaluated using two-way ANOVA followed by a post hoc Bonferroni multiple comparison test. Significant difference was observed in 10 *μ*g/mL paeonol group (*p* < 0.05), 50 *μ*g/mL paeonol group (*p* < 0.0001), and aspirin group (*p* < 0.0001) compared to control group. Post hoc analysis was performed at each concentration of collagen: ^*∗*^ *p* < 0.05 and ^*∗∗∗*^ *p* < 0.001 versus vehicle. The data in (b) was evaluated using the unpaired Student's *t*-test: ^*∗∗∗*^ *p* < 0.001 versus vehicle.

Paeonol, a main component of Moutan cortex that showed the potent suppression of the collagen-induced pressure rate among the 5 constituent crude drugs of KBG, was tested in a similar procedure (Figure 5). Factorial analysis indicated that there was a significant difference in 10 *μ*g/mL paeonol (*p* < 0.05), 50 *μ*g/mL paeonol (*p* < 0.0001), and aspirin (*p* < 0.0001) groups compared to control group. In post hoc analysis, paeonol (10–50 *μ*g/mL) inhibited the whole blood pressure rate increases induced by collagen (1–8 *μ*g/mL) in a concentration-dependent manner. The significant inhibitory effect of paeonol was observed at 50 *μ*g/mL, and the effect was equal to that of aspirin (100 *μ*M), a known platelet aggregation inhibitor (Figure 5(a)). The PATI values of paeonol (10–50 *μ*g/mL) increased in a concentration-dependent manner. The value at 50 *μ*g/mL of paeonol was equal to that of 100 *μ*M aspirin (Figure 5(b)).

## 4. Discussion

In the present study, we demonstrated that KBG inhibited the collagen-induced pressure rate increase and increased the PATI value in guinea pig whole blood, suggesting that KBG inhibited platelet aggregation.

To date, platelet aggregation has been evaluated commonly by turbidimetry using PRP [8]. This method relies only on platelet-platelet interactions. However, platelet aggregation* in vivo* is also mediated by interactions between platelets and leukocytes or erythrocytes and is controlled by chemical mediators from leukocytes, erythrocytes, and other types of cells [23, 24]. In the SFP method with guinea pig whole blood used in this study, platelet aggregation is measured as an increased filtration pressure of a whole blood sample caused by the clogging of microsieve formed through collagen-induced platelet aggregation. This method is considered an assay method under conditions closer to* in vivo* conditions compared with the PRP method. In addition to human blood, blood samples from various experimental animals, including mice, rabbits, guinea pigs, and dogs, are widely used for research on platelet function and development of antiplatelet aggregation agents. Physiological functions and drug responsiveness of platelets, however, have been suggested to vary from animal species. In this study, we chose the SFP method using guinea pig whole blood on the basis of a report that this method was more sensitive and suitable to test platelet aggregation than the PRP method [13].

In the present study, collagen-induced platelet aggregation (1–8 *μ*g/mL) was evaluated after 5 *μ*L of test substance solution dissolved in methanol was added to 195 *μ*L of whole blood. Alcohol is suggested to modify platelet functions [25]. However, in preliminary study, we confirmed in this experimental condition that there were no significant differences of the degree of collagen-induced platelet aggregation (1–8 *μ*g/mL) in the samples with or without methanol. In addition, as shown in Figure 5, we confirmed that aspirin inhibited collagen-induced platelet aggregation in the same condition. These verifications suggest that the experimental procedure used in the present study was appropriate to evaluate platelet aggregation function.

KBG, TSS, and KSS are known to have ameliorative effects on microcirculation disturbances. The latter two have been reported to show the platelet aggregation-inhibiting effects in PRP assays [5]. In the present study, these two formulations were used as positive controls and their inhibitory effects on platelet aggregation were confirmed by the SFP method. On the other hand, the detailed inhibitory effects of KBG on platelet aggregation remain still unclear, although there are some reports which suggested the antiplatelet aggregation effect [6] and some reports which suggested that factors such as the erythrocyte deformability and blood viscosity have been reported to be involved in its microcirculatory disturbances-improving effects [8]. In the present study, KBG demonstrated to suppress the collagen-induced whole blood pressure rate increase and increase the PATI value in tests using the SFP method with guinea pig whole blood (Figures 1 and 2), as well as TSS and KSS. An increase in the whole blood pressure rate in this assay system is believed to occur through platelet aggregation triggered by intracellular signal transduction of collagen-activated platelets, that is, activation of arachidonic acid cascade; the enhanced platelet aggregation leads to increased blood viscosity and eventually the pressure rate increases. Therefore, the suppressive effect on the pressure rate increase is presumably attributable to the inhibition of a certain step(s) in a series of events during collagen-induced platelet activation. As described above, platelet aggregation was observed with this assay method also for TSS and KSS, but not for RKT (a Kampo formulation for treatment of anorexia, dyspepsia, vomiting, gastritis, and stomach pain) [15–18] and SKT (a Kampo formulation for treatment of sudden muscle cramp and pain) [19–21] used as negative controls. These results suggest that the data obtained using the SFP method do not simply represent nonspecific reactions of Kampo formulations, which are combination formulas comprised of crude drugs. An enhanced level of platelet aggregation results in increased blood viscosity and impaired blood circulation. Various factors including increased blood viscosity, active oxygen species, reduced erythrocyte deformability, coagulation/fibrinolysis system, and adhesion factors are involved in the impaired blood circulation, that is, the increased resistance to blood flow in this study. Because reported effects of KBG include improved erythrocyte deformability and blood viscosity, it is conceivable that these factors are also involved in the suppression of increase in resistance to blood flow.

KBG comprises 5 constituent crude drugs: Moutan cortex, Cinnamomi cortex, Persicae semen, Paeoniae radix, and Hoelen. We tested the effects of individual crude drugs to identify the responsible crude drug for the platelet aggregation-inhibiting effects of KBG and found that Moutan cortex and Cinnamomi cortex had dose-dependent inhibitory activity (Figure 4(a)). This result suggests that these crude drugs may contain the active components.

Because we were able to obtain paeonol, a representative component of Moutan cortex that showed the potent activity, similar tests were conducted using paeonol. Like aspirin, paeonol significantly suppressed the collagen-induced whole blood pressure rate increase and significantly elevated the PATI value. This result was in agreement with the platelet aggregation-inhibiting effect by the PRP method [26]. Aspirin exerts the platelet aggregation-inhibiting effect by suppressing the collagen-activated arachidonic acid cascade through COX-1 inhibition. Paeonol has been reported to have a similar COX-1 inhibitory activity and antiradical activity. In addition, paeonol has also been reported to improve a microcirculatory disturbance induced by ischemia-reperfusion [27]. Although we did not test the Cinnamomi cortex-derived components, the cinnamic aldehyde has been reported to show an inhibitory effect on collagen-induced platelet aggregation [28]. Taken together, these findings suggest that Moutan cortex-derived paeonol and Cinnamomi cortex-derived cinnamic aldehyde are plausible active components responsible for the platelet aggregation-inhibiting effect of KBG, although more detailed studies are required to be conducted in the future on the active components and the mechanism of action.

In the adult, a 2.5 g dose of KBG, TSS, or KSS formulation is orally administered twice (5.0 g in total/day) or thrice (7.5 g in total/day) before meal or between meals in a day. It is difficult to compare effective dose or concentration directly between* in vitro* and* in vivo* studies, because the experimental conditions are different between them. To support the present results,* in vivo* verification study will be required in the future.

## 5. Conclusions

This study is the first to demonstrate, using the SFP method with guinea pig whole blood, the possibility that the platelet aggregation-inhibiting activity of Moutan cortex and Cinnamomi cortex is involved in the improving effects of KBG on impaired microcirculation and that the Moutan cortex-derived paeonol is partially responsible for these effects.
